# Videolaryngoscopy versus direct laryngoscopy for double-lumen endotracheal tube intubation in thoracic surgery - a randomised controlled clinical trial

**DOI:** 10.1186/s12871-020-01067-x

**Published:** 2020-06-16

**Authors:** Joachim Risse, Ann-Kristin Schubert, Thomas Wiesmann, Ansgar Huelshoff, David Stay, Michael Zentgraf, Andreas Kirschbaum, Hinnerk Wulf, Carsten Feldmann, Karl Matteo Meggiolaro

**Affiliations:** 1 Center of Emergency Medicine, University Hospital Essen, Hufelandstrasse 55, 45122 Essen, Germany; 2 Department of Anesthesiology and Intensive Care Medicine, Philipps-University Marburg, Baldingerstraße, 35033 Marburg, Germany; 3 Visceral, Thoracic and Vascular Surgery Clinic, University Hospital Giessen and Marburg GmbH, Baldingerstraße, 35033 Marburg, Germany

**Keywords:** Double-lumen endotracheal tube, Intubation, Thoracic Anaesthesia, Videolaryngoscopy

## Abstract

**Background:**

Double-lumen tube (DLT) intubation is necessary for thoracic surgery and other operations with the need for lung separation. However, DLT insertion is complex and might result in airway trauma. A new videolaryngoscopy (GVL) with a thin blade might improve the intubation time and reduce complexity as well as iatrogenic airway complications compared to conventional direct laryngoscopy (DL) for DLT intubation.

**Methods:**

A randomised, controlled trial was conducted in 70 patients undergoing elective thoracic surgery using DLT for lung separation. Primary endpoint was time to successful intubation. The secondary endpoints of this study were number of intubation attempts, the assessment of difficulty, any complications during DLT intubation and the incidence of objective trauma of the oropharynx and supraglottic space and intubation-related subjective symptoms.

**Results:**

65 patients were included (DL group [*n*  =  31], GVL group [*n*  =  34]). Median intubation time (25th–75th percentiles) in GVL group was 93 s (63–160) versus 74 (58–94) in DL group [*p* = 0.044]. GVL resulted in significantly improved visualisation of the larynx (Cormack and Lehane grade of 1 in GVL group was 97% vs. 74% in DL Group [*p* = 0.008]). Endoscopic examinations revealed significant differences in GVL group compared to DL group showing less red-blooded vocal cord [*p* = 0.004], vocal cord haematoma [*p* = 0.022] and vocal cord haemorrhage [*p* = 0.002]. No significant differences regarding the postoperative subjective symptoms of airway were found.

**Conclusions:**

Videolaryngoscopy using the GlideScope®-Titanium shortly prolongs DLT intubation duration compared to direct laryngoscopy but improves the view. Objective intubation trauma but not subjective complaints are reduced.

**Trial registration:**

German Clinical Trial Register DRKS00020978, retrospectively registered on 09. March 2020.

## Background

Lung separation is performed for thoracic surgery using several technical solutions (e.g. double-lumen intubation, bronchus blocker) [1]. Using a double-lumen tube (DLT) has become the most commonly used technique worldwide despite a more challenging procedure of tube placement compared to a conventional endotracheal tube [2–4]. Additionally, the rate of upper airway trauma including damage of pharynx, larynx (particularly vocal cords) and trachea during the insertion of a DLT using a conventional laryngoscope is significantly higher [3–5]. Reasons for this are a larger outer diameter and enhanced stiffness of a DLT compared to a conventional endotracheal tube. Due to the characteristics of a DLT, the direct view of the laryngeal structures are impaired during a DLT insertion [6, 7]. On the other hand, videolaryngoscopy has become standard for difficult airway management using conventional endotracheal tubes. In DLT intubation, the literature shows conflicting results of the benefits of videolaryngoscopy compared to direct laryngoscopy, especially intubation time as well as iatrogenic injuries [8, 9]. Moreover, meta-analysis data with moderate to low quality evidence exist, showing a higher success rate at first attempt, a higher incidence of malpositioned double-lumen tube and a lower incidence of oral, mucosal or dental injuries with videolaryngoscopy for DLT intubation [10].

A improved videolaryngoscopy device (GlideScope®-Titanium, Fa. Verathon Inc.) with a thinner blade (thickness of single use blade is 3 mm in size 3 and 2.7 mm in size 4) was introduced recently. A thinner blade design might be useful during the intubation of patients with a small oral cavity or limited mouth opening capacity. Presumably this device offers more space in the pharynx during DLT intubation than other videolaryngoscopy blades used in previous clinical trials which might result in improved intubation than previous videolaryngoscopy devices.

For GlideScope®-Titanium, single use blades are 3 mm (Size 3) and 2.7 mm (Size 4). Thus, we hypothesized that this improved GlideScope® videolaryngoscopy system (GVL) might result in a shortened intubation time and better visualisation of the anatomic structures compared with a conventional laryngoscopy approach for double lumen tube insertion. This could reduce the rate of airway trauma parameters and improve patient-centered outcome parameters.

## Methods

This prospective trial adheres to CONSORT guidelines and was approved by the local ethics committee (Ethikkommission Marburg, AZ115/16; 14.09.2016; retrospectively registered at the German Clinical Trials registry DRKS [DRKS00020978]). After written informed consent, adult patients scheduled for elective thoracic surgery requiring general anaesthesia with the need of a DLT for lung separation with American Society of Anesthesiologists physical status I–IV were enrolled from 23.02.2017 until 18.09.2017. Exclusion criteria were patient age < 18 years, non-elective surgery, pregnancy, scheduled rapid-sequence induction, contraindication for DLT insertion; contraindication to one-lung ventilation as well as abnormal physical status of the Cervical spine (e.g., after C-spine trauma, Bechterew’s disease).

### Primary and secondary endpoints

The primary endpoint of this study was duration of endobronchial DLT intubation (s). The intubation time was defined as: blade passes mouth opening → positive capnography (visualisation of 3 expirations in the capnography). The secondary endpoints of this study were number of intubation attempts, the assessment of difficulty and any complications during DLT intubation and the incidence of intubation-related injuries in both groups. Therefore, we performed two consecutive transnasal flexible endoscopic examinations (at the end of surgery and on the first postoperative day) of the oropharynx, of the supraglottic space and of the vocal cords, a follow-up survey by questionnaire and a dental examination to detect dental trauma.

### Sample size calculation

Sample size calculation was based on a previous study [7], which reported a mean (SD) time of 46 [11] s for DLT placement with videolaryngoscopy. Based on these results an a priori power analysis was performed for primary endpoint given a beta value of 0.80 and a significance level alpha of 0.05. We calculated a minimum required sample size of 29 patients per group to detect a 20% difference in the time taken for DLT intubation using non-parametric testing. Effect size of the duration of intubation used calculating sample size was 0.8 according to Cohen’s D. As a drop-out rate of 20% was assumed, the sample size was increased to 35 patients per group. Power analysis was performed using G*Power 3.1.9.6 for Mac OS X [11, 12].

### Randomization and allocation concealment

After written informed consent 70 patients were randomized via envelope method. Allocation concealment was achieved using sealed opaque envelopes. Performance blinding was not possible in this study design. Patients and postoperative outcome assessors (anesthetists, ENT specialist, dentist) were unaware of the randomization results. Statistical analysis was performed blinded to study allocation.

Patients were pre-medicated with 3.75–7.5 mg oral midazolam 45 min before surgery. On arrival in the induction area, all participants were blinded, randomly assigned to either direct laryngoscopy (DL) or videolaryngoscopy using the GlideScope® Titanium device (GVL, GlideScope; Verathon Inc., Bothell, WA) by sealed envelope randomisation. In the OR patients were positioned supine, standard monitoring was applied according to current national guidelines and peripheral intravenous access (IV) established. Patients received pre-oxygenation with 100% oxygen through a mask over 5 min. After pre-oxygenation, anaesthesia was induced with 0.3 μg kg^− 1^ sufentanil and 2 mg kg^− 1^ propofol intravenously. Thereafter, 0.6 mg kg^− 1^ rocuronium bromide was applied. The neuromuscular monitoring was performed by a relaxometry Train of Four (TOF). DLT intubation was performed when full relaxation status (TOF 0/4) was reached. Maintenance of general anesthesia was performed as total intravenous anaesthesia (TIVA) according to the local standards using propofol (4–6 mg kg^− 1^ h^− 1^) and remifentanil (15–25 μg kg^− 1^ h^− 1^) adjusted according to the measured anesthetic depth using Bispectral Index monitoring (BIS) at a target zone of 40–60.

**Fig. 1:**
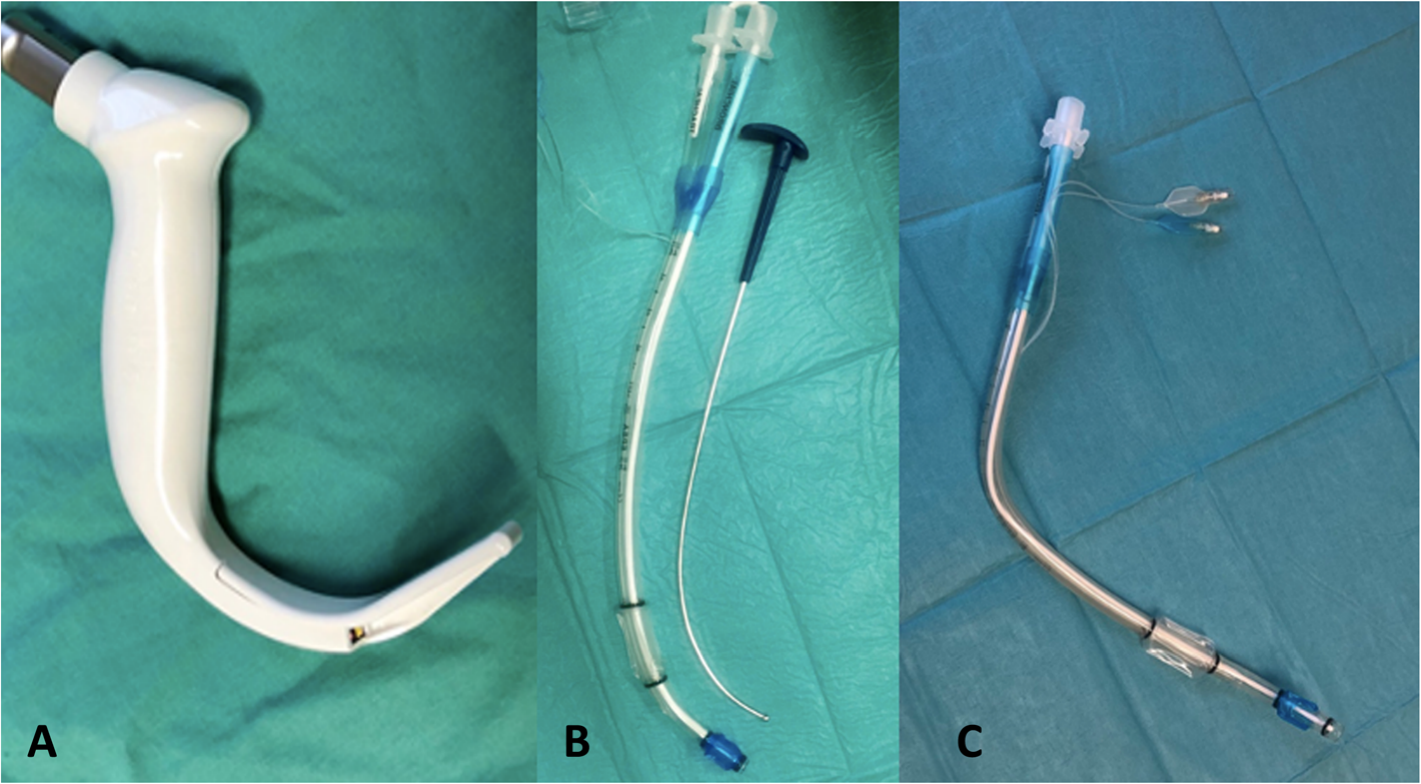
Comparison DLT used for intubation with GVL or DL. **a** GVL blade used; **b** DLT shaped for GVL; **c**) DLT shaped for DL

The size of the DLT (Rüsch Bronchopart; Teleflex Medical GmbH, Dublin, Ireland, 35–41 FR) used was determined for each patient according to the rule of Slinger et al. [13]. Intubation with a DLT was performed using a conventional MacIntosh blade (size 3 or 4) in the DL group or with the hyperangulated GlideScope®-Titanium Single-Use-blade (size 3 or 4) in the GVL group. The original DLT stylet was used for intubation in both groups. It was shaped according to the respective angulation of the blade used (Fig. 1). All intubations were performed by the same three experienced physicians.

### Postoperative assessment

The first endoscopic examination was performed at the end of surgery under general anesthesia and before extubation, while the follow-up endoscopic examination was performed the day after surgery under topical anesthesia. Stored endoscopic video clips were postprocessed for anonymisation and blinding. Thereafter, they were evaluated by three independent investigators (2 anaesthesiologists and 1 ENT specialist, investigator-blinded). The video clips of both examinations (postoperative & first postoperative day) were evaluated independently by blinded investigators. The hypopharynx, the vocal cords and the arytenoid cartilage were evaluated on the basis of various criteria. The different criteria were scored from according to the degree of injury (0 = not assessable, 1 = without pathological findings, 2 = minor injuries, 3 = severe injuries). The results were averaged for further analysis. Second, a physician of oral and maxillofacial surgery (investigator blinded) performed a dental examination after DLT intubation in all study patients, examining the patient for lip and dental trauma. Third, the patients first completed a questionnaire (Validated H&N35 Quality of Life Questionnaire Head and Neck Module and NRS) to express their subjective symptoms (hoarseness, etc.). The H&N Score ranged from 0 to 100. A high score correlated with a high degree of complaints and symptoms [14].

### Statistical analysis

All values for descriptive statistics and outcome parameters were non normally distributed. All non-normally distributed data were expressed as median and interquartile range (IQR). Dichotomous outcome parameters were expressed as events (percentages). Non-parametric data were analysed using the Mann-Whitney U-test. A *p* < 0.05 was considered being statistically significant. Normally distribution was assessed using Shapiro-Wilk test. Statistical analysis was performed using SPSS (IBM Corp. Released 2016, IBM SPSS Statistics for Windows, Version 24.0, Armonk, NY: IBM Corp.). Data are presented as tables and box-and-whisker diagrams.

## Results

### Demographics and biometric data

**Fig. 2:**
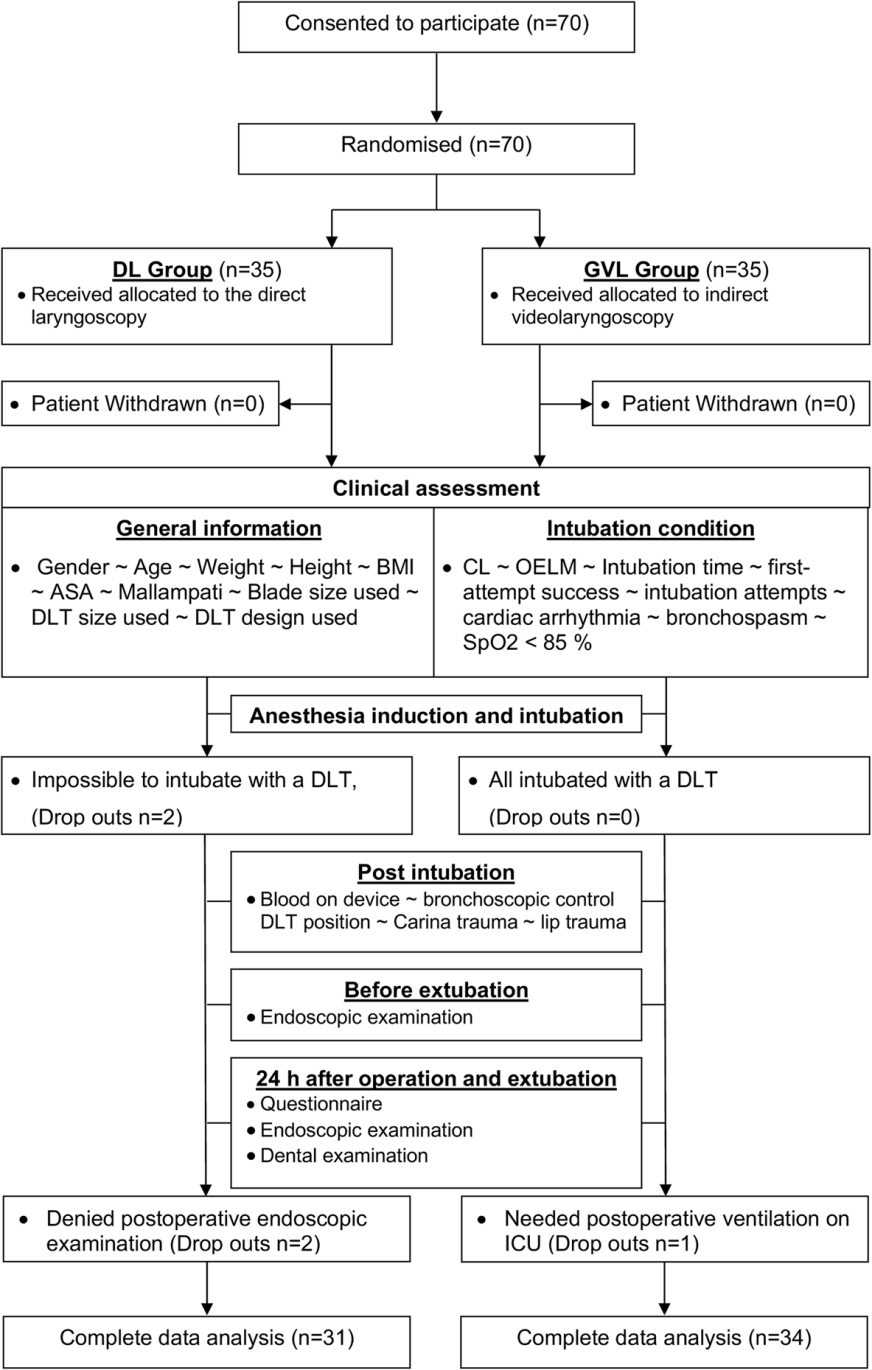
CONSORT Flow Diagram

After written informed consent, 70 patients were recruited. Out of 70 patients 65 completed the study and were included in the final intention-to treat analysis (Fig. 2). Four patients in the MacIntosh group (DL) and one patient in the GlideScope® group (GVL) were excluded from the final analysis. In two participants randomised to the DL group, the conventional DLT intubation attempts failed and the experienced examiner changed the method using videolaryngoscopy. Finally intubation with a single endotracheal tube and a bronchial blocker had to be performed in these two cases because of impossible DLT intubation, with both devices. Two participants randomised to DL group refused postoperative nasal endoscopic examination and one participant in GVL group needed long-term postoperative ventilation on the intensive care unit and was lost to follow-up. All five participants were excluded from the final analysis due to relevant study protocol violation as predefined (Fig. 2).

Both groups had no significant differences in biometric data and preoperative airway assessments (Table 1). In almost all cases, a left-sided DLT was used. It was noticeable that all three experienced specialists in thoracic anaesthesia who performed the DLT intubations in the study used smaller blades and tended towards smaller tube sizes in the GVL group. The difference between the two groups was statistically significant [*p* < 0.05] (Table 1).

**Table 1:** Biometric data and descriptive data of patients enrolled in the study. Data are presented as median (25th–75th percentile) or numbers (percentage), respectively

Parameter	DL (n 31)	GVL (n 34)	Mann Whitney U-test (*p*-Value)
**Gender (male/female)**	25/6	25/9	0.50
**Age (years)**	60 (52–65)	66 (58–75)	0.087
**Weight (kg)**	83 (75–95)	80 (68–90)	0.207
**Height (cm)**	178 (172–181)	173 (165–178)	0.038*
**Body mass index (kg m**^**−2**^**)**	25.7 (24.2–30.8)	25.2 (24.1–29.1)	0.604
**ASA** n (%)**:**			
I	0 (0%)	1 (3%)	0.824
II	10 (32%)	9 (26%)	
III	19 (61%)	24 (71%)	
IV	2 (7%)	0 (0%)	
**Mallampati score** n (%)**:**			
I	11 (35%)	14 (41%)	0.819
II	16 (52%)	14 (41%)	
> II	4 (13%)	6 (18%)	
**Blade size used** n (%)**:**			
size 3	1 (3%)	16 (47%)	< 0.001*
size 4	30 (97%)	18 (53%)	
**DLT size used** n (%)**:**			
35 French	1 (3%)	1 (3%)	0.023*
37 French	6 (19%)	18 (53%)	
39 French	17 (55%)	10 (29%)	
41 French	7 (23%)	5 (15%)	
**DLT design used** n (%)**:**			
left-sided	31 (100%)	33 (97%)	0.340
right-sided	0 (0%)	1 (3%)	

*Statistically significant

#### Primary endpoint

**Fig. 3:**
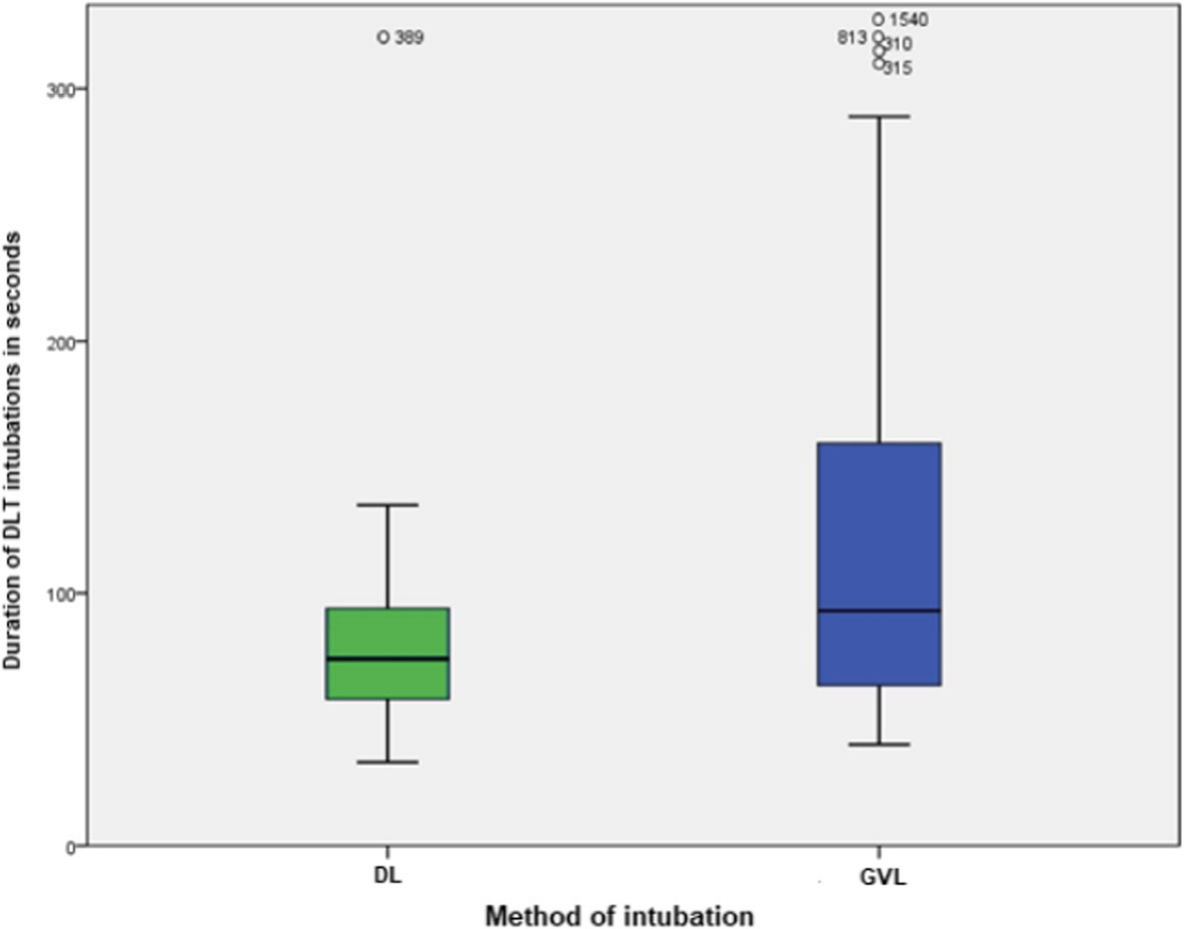
Duration of successful completion of DLT intubation between direct laryngoscopy (DL) and videolaryngoscopy (GVL). Legend:boxplot x-axis: methods Macintosh-DL (green) and GlideScope-VL (blue), y-axis: Duration of successful DLT intubation in seconds

Our primary endpoint duration of the successful completion of DLT intubation was significantly [*p* = 0.044] longer in the GVL group 93 s (63–160) compared to the DL group 74 (58–94) (see Table 2 and Fig. 3).

**Table 2:** DLT intubation data: Assessment of difficulty and complications. Data are presented as median (25th - 75th percentile) or number (percentage)

Parameter	DL (n 31)	GVL (n 34)	Mann Whitney U-test (*p*-Value)
**time to successful intubation (s)**	74 (58–94)	93 (63–160)	0.044*
**Cormack-Lehane score** n (%)**:**			
I°	23 (74%)	33 (97%)	0,008*
II°	7 (23%)	1 (3%)	
III°	1 (3%)	0 (0%)	
IV°	0 (0%)	0 (0%)	
**OELM maneuver** n (%)**:**			
yes	14 (45%)	11 (32%)	0,293
no	17 (55%)	23 (68%)	
**first-attempt success** n (%)**:**			
yes	28 (90%)	29 (85%)	0,287
no	3 (10%)	5 (15%)	
**DLT intubation attempts** n (%)**:**			
1	28 (90%)	29 (85%)	0,497
2	2 (7%)	2 (6%)	
3	1 (3%)	1 (3%)	
> 3	0 (0%)	2 (6%)	
**SpO2 < 85%** n (%)**:**			
yes	2 (6%)	2 (6%)	0,925
no	29 (94%)	32 (94%)	
**Bronchospasm** n (%)**:**			
yes	2 (6%)	0 (0%)	0,135
no	29 (94%)	34 (100%)	
**Cardiac arrhythmia** n (%)**:**			
yes	0 (0%)	0 (0%)	1,000
no	31 (100%)	34 (100%)	
**blood on device** n (%)**:**			
yes	4 (13%)	3 (9%)	0,599
no	27 (87%)	31 (91%)	
**Correct DLT position** n (%)**:**			
yes	24 (77%)	18 (53%)	0,041*
no	7 (23%)	16 (47%)	
**Carina trauma** n (%)**:**			
yes	0 (0%)	1 (3%)	0,340
no	31 (100%)	33 (97%)	
**Lip trauma** n (%)**:**			
yes	0 (0%)	0 (0%)	1,000
no	31 (100%)	34 (100%)	
**Dental trauma** n (%)**:**			
yes	0 (0%)	0 (0%)	1,000
no	21 (100%)	26 (100%)	
**Enamel fractures** n (%)**:**			
yes	0 (0%)	0 (0%)	1,000
no	21 (100%)	26 (100%)	

*Statistically significant

#### Secondary endpoints

Regarding the secondary endpoints our data showed better visualisation of the larynx with GVL. CL grade of 1 was with 97% more frequent in the GVL group. For the CL grade 1–4, a statistically significant difference in our data could be shown between the groups [*p* = 0.008]. In 32% of the patients in GVL group and 45% in the DL group, the OELM manoeuvre was necessary to achieve better conditions for endobronchial intubation [*p* > 0.05] (Table 2).

The first-attempt success did not differ significantly between GVL group (85%) and DL group (90%) [*p* > 0.05]. There was no statistically significant difference between both groups in the frequency of intubation attempts [*p* > 0.05]. None of the participants from DL group included in the analysis required more than three intubation attempts (Table 2).

During bronchoscopic control a correct DLT position directly after successful endobronchial intubation was reported in 77% of the CL group and only 53% of the GVL group. The difference observed between the two groups was statistically significant [*p* = 0.041] (Table 2).

There was no other significant difference in terms of direct complications under DLT intubation between the two groups (Table 2). Furthermore, lip and dental trauma, as well as enamel fractures examined by the dental follow-up, were not significantly different in both groups (Table 2).

When analysing the postoperative questionnaires (H&N35 and NRS Score) to record the subjective symptoms after DLT-Intubation, no significant differences were found between both groups [*p* > 0.05] (Tables 3 and 4).

**Table 3:** Results of relevant selected parameters from evaluation of the H&N35 Quality of Life Questionnaire Head and Neck Module (H&N Score). Data are presented as median (25th- 75th percentile)

Parameter (H&N Score)	DL (n 31)	GVL (n 34)	Mann Whitney U-test (*p*-Value)
**Sore throat**	0 (0–33)	0 (0–0)	0,402
**Dysphagia**	0 (0–0)	0 (0–33)	0,115
**Cough**	0 (0–33)	0 (0–33)	0,532
**Hoarseness**	0 (0–33)	0 (0–33)	0,640
**Dry mouth**	33 (0–100)	33 (0–67)	0,735
**Viscous mucus**	0 (0–67)	0 (0–33)	0,628
**Paresthesia**	0 (0–0)	0 (0–0)	0,340
**Language problems**	0 (0–0)	0 (0–0)	0,457
**Mouth opening problems**	0 (0–0)	0 (0–0)	1,000
**Toothache**	0 (0–0)	0 (0–0)	0,295

**Table 4:** Results of parameters additionally examined with numerical rating scale (NRS). NRS scores 1–3 correspond to mild, scores of 4–6 to moderate and scores ≥7 to severe symptoms. Values ​​are expressed as the number of patients or as the total number in percent

Parameter NRS Score	DL (n 31)	GVL (n 34)	U-test (*p*-Value)
**Sore throat** n (mild/ moderate/ severe) (total in %)	7/2/0 (29%)	6/3/1 (29%)	0,430
**Dysphagia** n (mild/ moderate/ severe) (total in %)	4/3/0 (23%)	6/3/2 (32%)	0,289
**Cough** n (mild/ moderate/ severe) (total in %)	9/3/1(42%)	10/3/1 (41%)	0,782
**Hoarseness** n (mild/ moderate/ severe) (total in %)	7/5/0 (39%)	4/6/1 (32%)	0,477

In contrast to the subjective symptoms, endoscopic examinations revealed significant differences in the GVL group compared to the DL group in the objectifiable trauma red-blooded vocal cord [*p* = 0.004], vocal cord haematoma [*p* = 0.022] and vocal cord haemorrhage [*p* = 0.002] (Table 5).

**Table 5:** Data of the reported intubation related injuries from two transnasal endoscopic examinations; before and 24 h after DLT extubation. All different criteria were scored from 0 to 3. (0 = not assessable, 1 = without pathological findings, 2 = minor injuries, 3 = severe injuries). Values ​​are expressed as median (25th - 75th percentile)

Parameter	DL pre-extubation	GVL pre-extubation	U-Test (***p***-Value)	DL 24 h post-extubation	GVL 24 h post-extubation	U-Test (***p***-Value)
**Vocal cord swelling**	1,00 (1,00-1,50)	1,00 (1,00-1,50)	0,310	1,33 (1,00-1,67)	1,33 (1,00-1,67)	0,478
**Vocal cord redness**	1,00 (1,00-1,33)	1,00 (1,00-1,00)	0,402	1,33 (1,00-1,33)	1,00 (1,00-1,00)	0,004*
**Vocal cord oedema**	1,00 (1,00-1,00)	1,00 (1,00-1,00)	0,309	1,00 (1,00-1,00)	1,00 (1,00-1,00)	0,589
**Vocal cord erythema**	1,00 (1,00-1,00)	1,00 (1,00-1,00)	1,0	1,00 (1,00-1,00)	1,00 (1,00-1,00)	0,624
**Vocal cord hematoma**	1,00 (1,00-1,00)	1,00 (1,00-1,00)	0,436	1,33 (1,00-1,67)	1,00 (1,00-1,33)	0,022*
**Vocal cord hemorrhage**	1,00 (1,00-1,50)	1,00 (1,00-1,00)	0,070	1,33 (1,00-1,33)	1,00 (1,00-1,00)	0,002*
**Vocal cord granuloma**	1,00 (1,00-1,00)	1,00 (1,00-1,00)	1,0	1,00 (1,00-1,00)	1,00 (1,00-1,00)	0,182
**Vocal cord mobility**	–	–	–	1,00 (1,00-1,00)	1,00 (1,00-1,00)	0,294
**Arytenoid cartilage trauma**	1,00 (1,00-1,00)	1,00 (1,00-1,00)	0,517	1,00 (1,00-1,00)	1,00 (1,00-1,00)	0,705
**Hypopharynx redness**	1,33 (1,00-1,33)	1,00 (1,00-1,42)	0,467	1,33 (1,33-1,67)	1,33 (1,00-1,50)	0,162
**Hypopharynx oedema**	1,00 (1,00-1,00)	1,00 (1,00-1,33)	0,149	1,00 (1,00-1,33)	1,00 (1,00-1,33)	0,433
**Hypopharynx hematoma**	1,00 (1,00-1,33)	1,0 (1,00-1,00)	0,323	1,33 (1,00-1,67)	1,00 (1,00-1,33)	0,226
**Hypopharynx hemorrhage**	1,00 (1,00-1,67)	1,17 (1,00-1,50)	0,895	1,00 (1,00-1,33)	1,00 (1,00-1,33)	0,777
**Subglottic redness**	–	–	–	1,00 (1,00-1,00)	1,00 (1,00-1,33)	0,072
**Subglottic oedema**	–	–	–	1,00 (1,00-1,00)	1,00 (1,00-1,00)	0,313
**Subglottic hematoma**	–	–	–	1,00 (1,00-1,50)	1,00 (1,00-1,67)	0,844
**Subglottic hemorrhage**	–	–	–	1,00 (1,00-1,00)	1,00 (1,00-1,33)	0,052

*Statistically significant

## Discussion

We compared the insertion of a double-lumen tube using a conventional MacIntosh laryngoscope with a thin blade videolaryngoscope. Intubation time was significantly prolonged for the videolaryngoscopy group. However, intubation conditions were improved but there were more malpositioned double-lumen tubes in the GVL group.

Despite an objective reduction in 3 of 17 predefined airway trauma parameters evaluated by follow-up endoscopy, patients in both groups showed comparable subjective wellbeing.

Prolonged intubation times for GVL videolaryngoscopy for DLT intubation were shown in previous studies [8, 15, 16]. Prolonged intubation times inevitably have a greater risk of hypoxia and could be harmful to patients with pulmonary comorbidities. In a prior study by Russell et al., anaesthetists found that GVL was more difficult to use than DL blade and DLT intubation took longer. In their study all DLT intubations were performed by less experienced anaesthetists [8], whereas in our study, all DLT intubations were performed by three consultants of anaesthesiology well experienced with DLT intubations in thoracic anaesthesia and GlideScope® videolaryngoscopy.

Contrary, a recent meta-analysis in 2018 by Liu et al., showed no difference in intubation time. The meta-analysis included studies with various videolaryngoscopes like Airtraq, McGrath Series 5, McGrath MAC - not all of these provide hyperacute angled devices [10]. Considering only the four studies using GlideScope® videolaryngoscopy included in the meta-analysis, our results are consistent with three of these four studies [8, 9, 15, 16]. Only Hsu et al. were able to show shorter intubation times with Glidescope videolaryngoscopy for DLT intubation [9]. From a clinical point of view, the intubation time differences between our groups are modest and potentially have no impact on morbidity but only demonstrate a more technically challenging situation using GVL instead of DL for DLT insertion.

The first attempt success rate reported here using GVL for DLT intubation was 85%. Our reported failure rate of 15% at the first attempt using GVL is similar to most of the results reported by other groups [8, 17, 18]. We were also unable to show a 100% first pass success rate with GVL, as showed by the group of Hsu et al. [9]. In the study of Hsu et al., all DLT intubations were performed by two experienced anaesthetists, who had both performed over 300 intubations using DLT with GVL. In addition, a external laryngeal manipulation was not required for successful DLT intubations with GVL [9].

The procedural step of advancing the DLT past the vocal cords seems to be the main sticking point for using hyper-angulated videolaryngoscopy [8, 19]. Multiple attempts may prolong the intubation time. Our findings support that DLT tube delivery and advancement into the trachea is the most difficult step in the procedure using videolaryngoscopy with hyper-angulated non-channelled blades for DLT intubations which causes prolonged intubation times for GVL. This assumption is supported by our data. The anaesthetists in our study needed a OELM manoeuvre in 32% of all DLT intubations with GVL and we reported significantly more malpositions of the DLT (in 47% of cases). Presumably caused by the rotation manoeuvre, which is needed for the advancement into the trachea, there is a higher incidence of main bronchus malposition of the DLT. Caused by the required bending of the DLT-tube for hyper-angulated blades, the tip of the DLT often hits the ventral wall of the trachea when advancing the tube past the vocal cords. Usually, a OELM manoeuvre is first performed to adjust the trachea, positioning it more posterior and more in line. Second, a rotation manoeuvre could be necessary. Such a rotation manoeuvre was described by Bustamante and Hernandez [20, 21]. Rotation manoeuvre more often results in the incorrect position of the DLT. Liu et al. concluded that the use of videolaryngoscopes, especially with a hyper-angulated blade for DLT intubation, complicates the already complicated DLT intubation technique through rotation manoeuvres [10]. Our data support the thesis that these sequential rotation manoeuvres are probably the reason why videolaryngoscopy increases the incidence of mispositioning of DLT. However, further studies are needed in the future to investigate this issue. We could not confirm our hypothesis that a thinner hyper-angulated blade provides better visibility and in consequence more space for a rotation manoeuvre and therefore a lower incidence of sore throat and hoarseness. Hsu et al. were able to show a lower incidence of sore throat and hoarseness [9]. There are controversial results in the literature. Russell et al. were unable to identify any significant differences in their study [8]. Due to the controversial results in the current literature and our results, the question remains whether the questionnaires used are sensitive enough to record differences in subjective symptoms.

With regard to the incidence of dental trauma, a study by Lee et al. (2011) comparing DL and GVL showed that less force is exerted on the teeth of the upper jaw when using GVL [22, 23]. This is in accordance with the results of the current study situation [8, 16]. Our dental follow up showed no significant differences in DLT intubation-related injuries. However, our study was not powered to detect that the incidence of dental trauma is different.

### Limitations

This study was randomised but has some limitations. First, the operators were not blinded to the intubation device used; however, it is difficult to circumvent this problem when evaluating different laryngoscopy devices. Nevertheless, the patient and follow-up endoscopic examinations were anonymised and blinded.

A further limitation of our study is the small number of patients with a supposed difficult airway (Mallampati 3 and 4, 13% in the DL group vs. 17% in the GVL group) and the low incidence of predicted difficult airways (CL 3 and 4, 3% in the DL group vs. 0% in the GVL group).

In addition, an appropriate rigid stylet for the DLT intubation with the GlideScope, like the GlideRite® Rigid Stylet, which is standardly used for the single lumen tube intubation, was not available at the start of the study [17]. Instead, we used the original rigid GlideRite® stylet for the single-lumen endotracheal tube as a template to shape the inner stylet of the DLT. A technique like the one developed and described by Bussier et al. and Bustamante et al. was not mandatory for our anaesthetists [17, 20].

DLT intubation with the GlideScope®-Titanium might be improved if the users are given some additional training and use the adequate rigid stylet for DLT, which keeps its shape and is better adapted to the hyper-angulated blade of the GVL.

There are currently many different videolaryngoscopes with varying designs and quality available on the market. For these reasons, our study results should not be generalised, and further investigation regarding videolaryngoscopy for double lumen tube insertion is needed.

## Conclusions

In conclusion, our randomized controlled trial showed faster intubation time using a conventional laryngoscope compared to the GlideScope® Titanium videolaryngoscopy for double-lumen tube insertion. Additionally, malposition of the DLT was more common in the videolaryngoscopy group. Despite some improvements in objectivable airway injuries during the postoperative course, there was no subjective difference for the patient as a relevant patient-centered outcome parameter. Further studies are needed to increase the number of first-pass optimal placement of DLT using videolaryngoscopy.

## Data Availability

The data that support the findings of this study are available from the corresponding author. The datasets used and analysed during the current study are available from the corresponding author on reasonable request.

## Abbreviation

BIS: Bispectral Index Monitoring
CL: Cormack and Lehane
DL: Direct Laryngoscopy
DLT: Double Lumen Tube
GVL: GlideScope Videolaryngoscopy
IQR: Interquartile range
NRS: Numeric Rating Scale
OELM: Optimal external laryngeal manipulation
OR: Operating room
SD: Standard deviation
TIVA: Total intravenous anaesthesia
TOF: Train of Four
